# Proceedings of Societies

**Published:** 1887-03

**Authors:** N. S. Hoff


### Proceedings of Societies.

The February meeting of the Odontological Society of Cincinnati was held at the residence of Dr. H. A. Smith, 126 Garfield Place, Tuesday evening, February 1st. The minutes of the previous meeting were read and approved.

The names of Drs. H. A. Smith, C. M. Wright, F. A. Hunter, F. W. Sage, Wm. Taft, C. R. Taft, Grant Mollyneaux, and A. T. Olmstead, having been regularly proposed at a previous meeting, were balloted for individually, and all unanimously elected members.

The names of Geo. W. Keely, O. N. Heise, and H. L. Moore were proposed for membership.

The committee appointed at the last meeting to prepare a programme to be submitted for the society's approval made a partial report, and were granted more time to perfect their programme, and instructed to provide a special programme for the March meeting.

Dr. J. Taft read a paper on Crown and Bridge Work, with especial reference to the treatment necessary to render diseased roots capable of sustaining crowns. He also exhibited an apparatus, designed by Dr. J. J. R. Patrick, of Illinois, for stamping metal crowns and adapting them to the teeth. The paper was an interesting one, and the subject, including the various cements used for setting the crowns, was variously discussed by all present.

At this point in the proceedings Dr. Smith stated that if the members of the society would adjourn to an upper room of his home they could have an opportunity to make a practical demonstration in the art of *crown insertion*, which, although it was not new, had from the remotest ages proved eminently satisfactory.

The place of adjournment proved to be our host's elegant and commodious dining room, where the bountiful feast and the enduring cement of professional fellowship proved a powerful agent for the removal of all professional pathological conditions, and made the process of *crown setting* one of substantial benefit as well as an occasion of good cheer.

The society then adjourned to meet March 29th at the residence of Dr. A. Berry. N. S. Hoff, *Secretary.*

